# Long term survivors with metastatic pancreatic adenocarcinoma treated with gemcitabine: a retrospective analysis

**DOI:** 10.1186/1756-8722-2-13

**Published:** 2009-03-16

**Authors:** Bernardo HL Goulart, Jeffrey W Clark, Gregory Y Lauwers, David P Ryan, Nina Grenon, Alona Muzikansky, Andrew X Zhu

**Affiliations:** 1 Division of Hematology/Oncology, Biostatistics Center, Massachusetts General Hospital, Harvard Medical School, Boston, MA, USA; 2 Department of Pathology, Biostatistics Center, Massachusetts General Hospital, Harvard Medical School, Boston, MA, USA; 3 Biostatistics Center, Massachusetts General Hospital, Harvard Medical School, Boston, MA, USA; 4 Massachusetts General Hospital Cancer Center, 55 Fruit Street, POB 232, Boston, MA, USA

## Abstract

**Background:**

Metastatic pancreatic adenocarcinoma has a short median overall survival (OS) of 5–6 months. However, a subgroup of patients survives more than 1 year. We analyzed the survival outcomes of this subgroup and evaluated clinical and pathological factors that might affect survival durations.

**Methods:**

We identified 20 patients with metastatic or recurrent pancreatic adenocarcinoma who received single-agent gemcitabine and had an OS longer than 1 year. Baseline data available after the diagnosis of metastatic or recurrent disease was categorized as: 1) *clinical/demographic data *(age, gender, ECOG PS, number and location of metastatic sites); 2) *Laboratory data *(Hematocrit, hemoglobin, glucose, LDH, renal and liver function and CA19-9); 3) *Pathologic data *(margins, nodal status and grade); 4) *Outcomes data *(OS, Time to Treatment Failure (TTF), and 2 year-OS). The lowest CA19-9 levels during treatment with gemcitabine were also recorded. We performed a univariate analysis with OS as the outcome variable.

**Results:**

Baseline logarithm of CA19-9 and total bilirubin had a significant impact on OS (HR = 1.32 and 1.31, respectively). Median OS and TTF on gemcitabine were 26.9 (95% CI = 18 to 32) and 11.5 (95% CI = 9.0 to 14.3) months, respectively. Two-year OS was 56.4%, with 7 patients alive at the time of analysis.

**Conclusion:**

A subgroup of patients with metastatic pancreatic cancer has prolonged survival after treatment with gemcitabine. Only bilirubin and CA 19-9 levels were predictive of longer survival in this population. Further analysis of potential prognostic and predictive markers of response to treatment and survival are needed.

## Background

Pancreatic cancer is the fourth leading cause of death in men and fifth in women in the United States [1]. An estimated 37,680 new patients and approximately 34,290 deaths are expected to occur from this disease in 2008. It has a poor prognosis with less than 5% of all patients alive 5 years after diagnosis [2]. Surgical resection remains the only curative approach. Unfortunately, by the time of diagnosis, only 15% of patients have resectable tumors. Within this group, the 5-year survival is 20–25% [2,3]. The vast majority of patients present with locally advanced or metastatic disease. For the metastatic group, the overall survival (OS) ranges between only 3 to 6 months [4-6]. At present, palliative chemotherapy is considered the treatment of choice for patients with metastatic disease and good performance status. Prior to 1996, standard chemotherapy usually consisted of 5-fluorouracil (5-FU) based regimens, with small impact on OS [7-10].

Gemcitabine (difluorodeoxycytidine; dFdCA) is a nucleoside analog with broad antitumor activity. When compared to 5-FU in a randomized phase III trial, there was a modest improvement in OS (5.6 *vs*. 4.4 months) as well as a higher rate of clinical benefit (24% to 5%) favoring gemcitabine [11]. Consequently, gemcitabine has become the standard of care for metastatic or recurrent adenocarcinoma of the pancreas. Studies over the past 10 years have tried to improve on single agent, standard 30-minute gemcitabine therapy. A randomized phase II trial between standard 30-minute infusion of gemcitabine against a fixed dose-rate infusion of 10/mg/m^2^/min of the same drug revealed a significant benefit in 1 and 2-year OS for the prolonged infusion rate [12]. Several trials have evaluated whether there is any benefit for gemcitabine-based combinations, including molecular targeted agents, over gemcitabine alone. Although several of these have shown a higher response rate favoring the combined regimens, a clear benefit in OS has yet to be shown [13-16]. Despite the benefit of gemcitabine, most patients with advanced disease still do poorly, with a median Time-to-Tumor Progression (TTP) between 2 to 3 months and median OS of 5 to 6 months. The one-year OS varies from 2 to 32% in the most recently published series [11,13,17]. Such a broad variation can be partially explained by the fact that some of these studies have also included patients with locally advanced disease, with median survival ranging from 9 to 12 months. However, those results may also suggest that a subgroup of patients with metastatic and/or recurrent disease may experience prolonged survival when treated with gemcitabine. Whether these patients benefited from treatment or represented a selected population with favorable prognosis is unknown.

The identification of potential predictors of long-term survival in patients with metastatic pancreatic cancer can help physicians in the decision-making process for individual patients. Once identified, these factors could also be useful in designing future clinical trials. In an attempt to assess the clinical and pathological predictors of prolonged survival, we performed a systematic review and outcome analyses of patients with adenocarcinoma of the pancreas who survived more than one year from the diagnosis of metastatic or recurrent disease and received gemcitabine.

## Methods

This systematic review received the approval of the local Institutional Review Board. All information regarding the patients included in the study remained confidential during and after data collection.

We searched for patients with metastatic or recurrent pancreatic adenocarcinoma diagnosed from March 1996 to July 2002 who were treated with gemcitabine in the outpatient clinic at Massachusetts General Hospital Cancer Center. Patients and chemotherapy regimens were queried through the institutional tumor registry database. We selected the following inclusion criteria: histopathological diagnosis of pancreatic adenocarcinoma based on the reports and confirmed on an independent review of pathological specimens; metastatic disease at presentation or recurrent disease after surgery or chemoradiation for local or locally advanced disease, respectively; a survival longer than 12 months from the initial diagnosis of metastatic or recurrent disease; palliative treatment with gemcitabine. Patients could have received additional chemotherapy or experimental therapy after failure or intolerance to gemcitabine. We adopted the following exclusion criteria: histopathological type other than adenocarcinoma, periampullary cancers, other active cancers, brain metastasis as the single metastatic site, patients not treated with gemcitabine, or survival shorter than 12 months from the initial diagnosis of metastatic disease or from documentation of recurrence. Patients did not need to have measurable disease to be enrolled.

A systematic review of the charts was performed. We searched for baseline information present at the time of the diagnosis of metastatic or recurrent disease. The only data evaluated during gemcitabine treatment was the serum CA19-9 level. When patients underwent surgery as the initial treatment before recurrence, the pathological report and material of the procedure were also reviewed. The data were grouped and analyzed in four major categories:

### 1) Demographic/clinical data

Patients' age, gender, ECOG PS, number of metastatic or recurrent sites, and location of metastatic/recurrent disease.

### 2) Laboratorial data

Baseline blood tests such as hematocrit (hct), hemoglobin (hgb), renal and hepatic functions, LDH, glucose, CA19-9, and lowest CA19-9 value during treatment with gemcitabine or combinations.

### 3) Pathological data

In all cases, the available diagnostic material was reviewed to confirm that all tumors were adenocarcinoma. The degree of differentiation (well, moderate, and poor) was recorded. For the resected cases, the size of the tumor, marginal status and number of positive nodes were also collated.

#### 4) Outcomes data

We determined the median OS, 2-year OS, and median time to treatment failure (TTF) on gemcitabine as well as the median number of chemotherapeutic/investigational regimens. Survival is shown as medians with 95% confidence intervals (95% CI). Response rates according to serial CT scans and CA 19-9 levels were determined. We analyzed CT scan responses based on modified WHO criteria to define a response [18]. CA 19-9 responses were defined as a ≥ 50% drop from the baseline level seen in any measurement during gemcitabine treatment. For both CT scan and CA 19-9 methods, we compared the OS of responders *vs*. non-responders by non-paired student's t-test.

A univariate regression analysis was undertaken to examine any potential factors that might impact on OS. As we did not detect more than two significant factors, we did not proceed with a multivariate analysis. The Cox proportional hazard model was used for all variables [19]. OS curves were obtained through the Kaplan-Meyer method [20]. Alive subjects were censored by the last medical visit. Death dates were available in the charts or through the web site page .

## Results

From March 1996 to September 2002, we identified 435 patients with adenocarcinoma of the pancreas with either distant metastasis at diagnosis or recurrent cancer after surgical resection or progressive disease after chemoradiation for locally advanced disease. Of these, 22 patients had an OS longer than 12 months. Two patients were excluded because one was diagnosed with a periampullary tumor and the other one had a histological diagnosis of mucinous cystadenocarcinoma. Therefore, 20 patients were included in the final analysis.

All patients received gemcitabine given as single-agent. *Baseline characteristics *were defined as those measured at the time metastatic or recurrent disease was first documented. In the case of CA19-9 levels, we analyzed both the baseline and lowest values during gemcitabine treatment.

### 1) Demographic/clinical Data

Demographic and clinical data are shown in Table 1. Median age at baseline was 59 years. Sixty-five percent of patients were males and 35% females with a male:female ratio of 1.85:1.0. The patients had a baseline ECOG PS of 0, 1 or 2 in 30, 60 and 10% of the cases, respectively. As expected, none of the individuals had a baseline PS of 3 or 4. Interestingly, of the two patients with PS 2 at baseline, one has shown an OS of 29.8 months and the other is still alive at 13 months of follow-up, although he has had disease progression on gemcitabine. Seventy-five percent of the individuals had only one metastatic or recurrent site at baseline, while the remaining 25% presented with 2 sites. None of the patients presented with more than 2 metastatic or recurrent sites.

**Table 1:** Clinical characteristics (n = 20)

	Values	HR	p value
Median age (Yr)	59 (38 – 72)	1.04	0.16

Gender			

M	65%	0.89	0.85

F	35%		

ECOG PS (%)		1.04	0.92

0	30		

1	60		

2	10		

3 & 4	0		

N° of Sites		0.80	0.74

1	75%		

2	25%		

>2	0%		

Initial Sites		0.99	0.99

liver	15%		

lung	10%		

peritoneal	20%		

local	25%		

nodal	0%		

bone	5%		

combinations	25%		

Median age was determined at diagnosis of metastatic disease or at documentation of recurrence. M = male; F = female; ECOG PS Performance Status according to Eastern Cooperative Oncology Group; NO of Sites = number of metastatic sites at diagnosis of metastatic disease or recurrence; Initial Sites = Proportion of initial metastatic/recurrent sites according to the involved organ. HR = Hazard ratio for Overall Survival after univariate analysis. P value = level of significance.

The initial sites of metastasis or recurrence were considered individually for subjects who had only one site. Those with 2 sites were grouped together under the designation of "combination". Among the patients with single site of metastasis or recurrence, 25% (n = 5) presented with local recurrences or progression, 20% (n = 4) with peritoneal carcinomatosis, 15% (n = 3) with liver metastasis, 10% (n = 2) with lung metastasis and 5% (n = 1) with bone metastasis only. Of the 5 patients with more than one metastatic site, 4 had a nodal recurrence together with another site: 2 with local recurrences, 1 with liver and 1 with lung metastasis. Only one patient had a combination of liver and peritoneal metastases.

### 2) Laboratory data

A summary of the tests analyzed is shown in Table 2. Most patients had normal hepatic and renal function. The median hematocrit level of 37.8% was slightly below the normal range. Several individuals showed increased levels of transaminases at baseline, but none exceeded 2.4 and 2.2 times the upper normal limits for SGOT and SGPT, respectively. Alkaline Phosphatase (Alk. Ph.) varied from 58 to 434 U/L, with a median value of 111.5 U/L. Ten patients (50%) demonstrated elevated levels (118 to 434 U/L) at baseline. Total bilirubin levels ranged from 0.2 to 14.6 mg/dL with a median value of 0.65 mg/dL. Five subjects (25%) had elevated bilirubin levels at baseline, ranging from 1.4 to 14.6 mg/dL. Two patients with bilirubin values of 1.7 and 5.0 mg/dL, respectively, were not treated with a biliary drainage procedure at baseline. The other 3 patients had their biliary tree decompressed surgically (1 patient) or by stent placement (2 patients). The time interval from the biliary drainage procedure to baseline bilirubin assessment was 3 weeks in 2 cases and 4 weeks in one case. Of note, the patient with the baseline bilirubin of 14.6 mg/dL had a successful stent placed 3 weeks earlier, when his bilirubin levels measured 21.9 mg/dL. None of the individuals presented with severe anemia at baseline. This is highlighted by the lowest hct and hgb levels of 30.7% and 10.4 g/dl, respectively. Serum glucose levels were in the upper normal limit or slightly elevated in all but one patient with diabetes mellitus who had baseline glucose level of 247 mg/dL. The median value for LDH was also in the normal range, although it was increased in 6 individuals, from 214 to 1116 U/L.

**Table 2:** Laboratory values

Tests	Reference	Median	Range	HR	p value
HCT (%)	41–53	37.80	30.70 – 44.6	1.03	0.73

					

Hgb (g/dL)	13.5–17.5	13.20	10.40 – 15.5	1.14	0.59

					

Bilt (mg/dL)	0–1	0.65	0.20 – 14.6	1.31	0.02*

					

SGOT (mg/dL)	10–40	37.50	17 – 97	0.99	0.79

					

SGPT (mg/dL)	10–55	28.50	12 – 121	0.98	0.21

					

AP (U/L)	45–115	111.50	58 – 434	1	0.93

					

GLU (mg/dL)	70–110	114	69 – 247	0.99	0.88

					

BUN (mg/dL)	8–25	13.50	8 – 25	0.96	0.51

					

Cr (mg/dL)	0.60–1.50	0.80	0.50 – 1.90	0.49	0.42

					

LDH (U/L)	110–210	196.50	69 – 1116	0.99	0.07

					

CA19-9 (U/mL)	37	360	11 – 38000	1	0.28

					

log CA19-9 (U/mL)	-	2.55	1.04 – 4.57	1.32	0.04*

					

log CA19-9 dif (U/mL)	-	5.81	1.38 – 10.50	1.15	0.34

HCT = hematocrit; Hgb = hemoglobin; Bilt = total bilirubin; SGOT = aspartate transaminase; SGPT = alanine transaminase; AP = Alkaline Phosphatase; GLU = glucose; BUN = Blood Urea Nitrogen; Cr = creatinine; LDH = Lactic dehydrogenase; CA19-9 = tumor marker; log CA19-9 = logarithm of CA19-9; log CA19-9 dif = difference between the logarithm of pre- and post-therapy CA19-9 levels. HR = Hazard ratio for Overall Survival after univariate analysis. P value = level of significance.

Tumor marker CA19-9 demonstrated a broad variation in the baseline values (from 11.0 to 38,000 U/L). As it showed an exponential distribution, we also expressed the values as the logarithm of CA19-9. The two individuals with the highest CA19-9 values, 25,580 and 38,000, had an OS of 12.0 and 21.6 months, respectively. The former was alive at the time of this analysis.

### 3) Histopathologic data

Table 3 summarizes the histopathologic characteristics. Six patients had undergone Whipple surgery, 6 had been diagnosed by fine needle aspiration (FNA) and 8 by biopsies. In all but 2 cases (2 biopsies), the pathologic material could be reviewed. A diagnosis of adenocarcinoma was confirmed in all cases. In the 2 cases for which we could not review the tissue, a diagnosis of adenocarcinoma had been confirmed on site before the material was returned to the referring institution.

**Table 3:** Pathologic characteristics

Features	Values	HR	p value
Adenocarcinoma (n = 20)	100%	NA	NA

Grade (n = 12)		1.22	0.78

well	8%		

mod	42%		

Poorly	50%		

Margins status (n = 6)		NA	NA

negative	34%		

positive	66%		

median nodes (n = 6)	4 (1 – 7)	NA	NA

Margins and nodal status were reported only for the cases initially treated with surgery (Whipple's procedure). Grade status was reported in 12 cases. Description: n = number of subjects; well = well differentiated; mod = moderately differentiated; poorly = poorly differentiated. HR = Hazard ratio for Overall Survival after univariate analysis. P value = level of significance.

**Figure 1:**
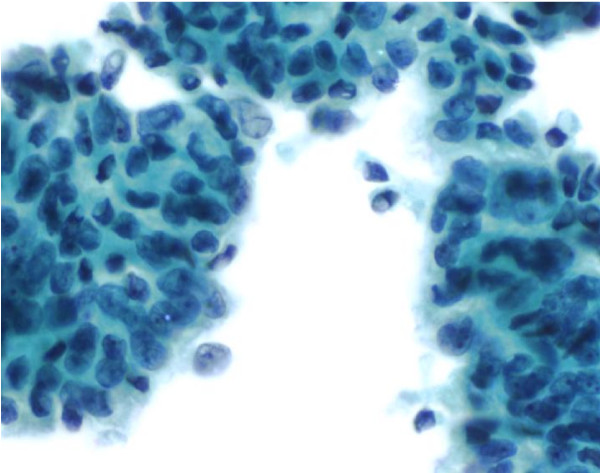
**Cytology from one FNA showed a moderately differentiated ductal adenocarcinoma**. It was characterized by a cohesive group of malignant cells showing nuclear crowding and overlapping. Minimal nuclear irregularity and prominent nucleoli can be seen (Papanicolaou 40× per High Power Field).

**Figure 2:**
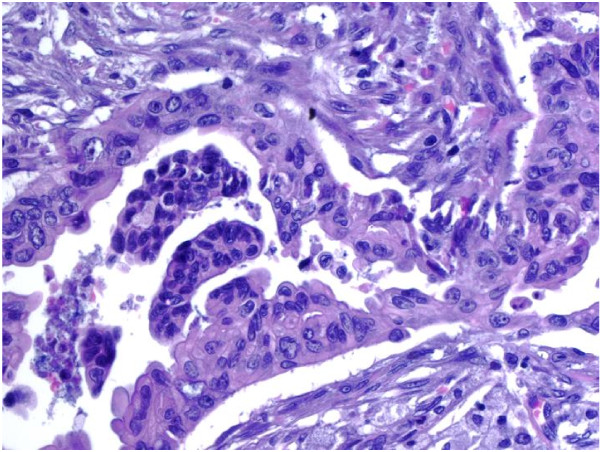
**Pathology from one Whipple specimen showed ductal adenocarcinoma, moderately differentiated**. Irregularly shaped malignant glands infiltrated the desmoplastic stroma. Marked nuclear atypia was observed (Hematoxylin and Eosin 40× per High Power Field).

Among the 6 resected cases, the size of the tumor was available in 5. Only one case measured less than 3 cm (2.5 cm) while the other 4 had a median size of 4.3 cm (range: 3.3 to 6.5 cm). Pathological margins and nodal status were assessed for the 6 surgical procedures. Of these, status of the resected margins was reported as negative in two cases (34%) and positive in four cases (66%). Five of the 6 resected patients had metastatic lymph nodes. The number of positive lymph nodes ranged from 1 to 7, with a median of 4. Excluding the cytologic material, the degree of differentiation was evaluated in 12 patients. One case (8%) showed a well-differentiated tumor, as opposed to five cases (42%) of moderately differentiated and 6 cases (50%) of poorly differentiated tumors. The subject with the well-differentiated tumor had a survival of 57.9 months, while subjects with poorly differentiated tumors showed a survival range from 12.4 to 24 months. An example of the cytology sample from FNA and a pathology specimen from Whipple surgery were shown in Figures 1 and 2, demonstrating the diagnosis of adenocarcinoma.

### 4) Outcomes data

**Figure 3:**
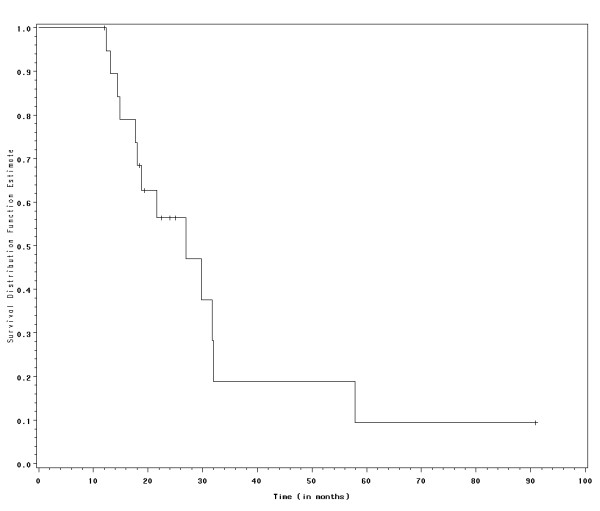
**Survival curve according to Kaplan-Meier method**. Individuals alive at the time of the study were censored and represented as dashes on the curve.

Table 4 summarizes the survival data. The median OS for all patients was 26.9 months (95% CI = 18 – 32 months) and the 2-year OS rate was 56.4%. Two patients had a prolonged OS of 57.9 and 90.9 months, respectively, and the last one was alive at the time of the present report. The Kaplan-Meier curve for OS is shown in Figure 3. All patients were initially treated with single-agent gemcitabine.

**Table 4:** Clinical outcomes

	Values	95% CI
Median TTF Gem (mo)	11.50	9 – 14.3

Median OS (mo)	26.90	18 – 32

OS 2 yr (%)	56.40	NA

N° of chemo lines		

Median	1 (1 – 6)	NA

TTF Gem = Time to Treatment Failure on gemcitabine; OS = Overall Survival; OS 2 yr = 2-year Overall Survival; No of chemo lines = Number of chemotherapeutic lines; 95% CI = 95% Confidence Interval.

The median TTF on gemcitabine was 11.5 months (95% CI = 9.0 – 14.3). These results contrast substantially with the usual TTF of 2 to 3 months on gemcitabine for non-selected populations with metastatic pancreatic cancer. However, TTF on gemcitabine varied significantly among these patients ranging from 5.7 to 50.8 months. Interestingly, the subject with the shortest TTF (5.7 months) had the longest OS (90.9 months). On the other hand, the individual who survived for 57.9 months spent 50.8 months on gemcitabine. Together, these results suggest that, even among patients with prolonged survival, the presumed benefit from gemcitabine can differ significantly.

All 20 patients had serial CT scans available for response analysis. Five patients (25%) had an objective radiological response. Their median OS was 24.0 months, compared to an OS of 18.8 months for non-responders (p = 0.66). Of 19 patients with CA 19-9 levels available, 12 (63%) showed a tumor marker response. As shown in Table 5, the median OS of responders was 20.5 months, compared to a median OS of 26.9 months for non-responders (p = 0.45.). Of the 5 patients with a radiological response, 4 (80%) also had a tumor marker response. Five patients were additionally followed by PET scans. All of them showed some reduction of tumor uptake of ^18^Fluoro-deoxy-glucose (FDG), and two patients had a complete normalization of PET scans. None of the patients with a PET response obtained a CT scan response. All of the patients with PET responses showed a CA 19-9 response.

**Table 5:** Response rates and correlation with survival according to CT scans and CA 19-9.

	Responses (%)	Median OS responders	Median OS Non-responders	p value
CT scans	5 (25%)	24 mo	18.8 mo	0.66

CA 19-9	12 (63%)	20.5 mo	26.9 mo	0.45

CT scans = Computerized Tomography scans; Median OS = Median Overall Survival; mo = months. CT scan responses are based on modified WHO criteria. CA 19-9 responses were determined by a 50% drop in the baseline CA19-9 value. p value = level of significance.

The number of systemic treatment regimens ranged from 1 to 6, with a median number of 1. If we consider only the patients who received more than one chemotherapy line (50%), the median number of treatments was 3. There was no significant difference in the overall survival of patients treated with one line of chemotherapy compared with patients treated with 2 or more lines (median OS 18 vs. 24 months, HR = 0.84; p = 0.29).

Gemcitabine was well tolerated in this population. However, we observed two cases of hemolytic uremic syndrome (HUS) (10%). They occurred 6 and 8 months after the onset of gemcitabine treatment, respectively. The first patient survived for 14 months after the diagnosis of HUS, and died of disease progression with an overall survival time of 26.9 months. The second patient is still alive 15 months after the diagnosis of HUS. His renal function has significantly improved and he is left with a slight residual decrease in his renal function.

### 5) Univariate Analysis

Using the Cox proportional hazard model, we tested all variables for significant impact on OS (Table 2). Only log CA19-9 at baseline and total bilirubin (bilt) had a negative impact on OS (HR = 1.32; p = 0.044 and HR = 1.31; p = 0.021, respectively). There was no statistical significant association between Log CA19-9 response during gemcitabine and OS (HR = 1.15; p = 0.34).

Potential prognostic factors such as ECOG-PS, positive margins, histologic grade, and nodal status of the initial surgical specimen as well as number of initial metastatic sites did not demonstrate a significant impact on OS [21,22]. The only patient with a well-differentiated adenocarcinoma had a prolonged survival of 57.9 months. The median OS of 6 patients with well and moderately differentiated tumors was 27.4 months, compared to a median OS of 18.9 months of 6 patients with poorly differentiated tumors (p = 0.106, using a non-paired student's t-test).

## Discussion

A subgroup of patients with metastatic or recurrent pancreatic cancer have outcomes that are significantly better than the average patient population. This study sample represents only approximately 5% of patients treated with palliative gemcitabine at this institution. This proportion of long-term survivors is relatively low when compared with most randomized clinical trials of gemcitabine [11-17]. Potential explanations for this low 1-year survival rate include incomplete ascertainment of long-term survivors, an overall worse prognosis of patients referred for treatment at our institution (referral bias), and the selection of patients with better performance status in clinical trials (selection bias). It is also possible that some patients from our initial population (n = 435) never received gemcitabine due to rapid tumor progression.

The median OS and TTF on gemcitabine were 26.9 and 11.5 months, respectively, while most of the randomized trials with single agent or gemcitabine-based combinations report OS and TTP between 3.8 to 6.7 months and 2.2 to 3.5 months, respectively [13-16]. The striking differences in survival outcomes between this group of patients and patients on randomized trials suggest two possible explanations: 1) selected patients receive significant benefit from gemcitabine or 2) this selected population has a better prognosis independent of treatment modality. Clearly, some combination of these might also be true. Given the retrospective nature of this analysis, no firm conclusion differentiating these two possibilities can be made. However, several findings suggest that treatment with gemcitabine accounts, at least in part, for the prolonged survival of these patients. The 25% response-rate to gemcitabine seen in this study is considerably higher than the 5–10% described in the literature [11-13,15]. Although the differences in OS between CT scan responders and non-responders (24 and 18.8 months, respectively) did not reach statistical significance, there was a trend towards longer survival in responders. The TTF on gemcitabine for the entire group of patients was 11.5 months, significantly longer than that in unselected patients. However, this retrospective study does not allow us to attribute the observed long-term outcomes to either increased gemcitabine responsiveness in selected patients or the presence of prognostic factors associated with prolonged survival.

There were no clinical characteristics that predicted long-term survival within this group. Interestingly, commonly considered prognostic factors in metastatic pancreatic cancer did not have a significant impact on OS in our analysis. ECOG PS, a significant clinical factor for OS in previous studies, was not correlated with survival [5,21,22]. This was somewhat expected, because there were no patients with a PS greater than 2 in this study. Likewise, age, gender, number and location of initial sites of metastasis, and pathologic grade did not correlate with OS. The presence of liver metastasis is also considered a poor prognostic factor. The fact that only 3 patients (15%) had liver metastasis at presentation could have contributed to the relative prolonged survival outcomes of our study sample.

The only two factors that significantly influenced OS were total bilirubin and the serum log CA19-9 at baseline in our study. Elevated serum bilirubin had a negative impact on OS, with a HR = 1.31 (p = 0.021). Elevated total bilirubin probably reflects the severity of initial biliary obstruction, incomplete drainage from biliary decompression procedures, hepatic dysfunction due to prolonged cholestasis or the presence of liver metastasis. Cholestasis due to tumor biliary obstruction only partially explains the detrimental contribution of serum bilirubin on OS, as 3 of the 5 patients with elevated bilirubin had a successful biliary decompression procedure. Since only 5 patients had elevated bilirubin levels, the association of bilirubin levels and overall survival should be interpreted with caution. CA19-9 levels correlated with OS, although in a logarithmic rather than in a linear fashion. The decrease in CA19-9 values after gemcitabine did not correlate with the length of OS in our study. Since the patients included in this analysis were selected for at least one year survival after initiation of gemcitabine therapy, and the group as a whole had a relatively high proportion of CA19-9 responders (63%), it is perhaps not surprising that there was not a detectable effect of CA19-9 response on duration of survival.

Previously, *Heinemann et al *described a correlation of decreasing levels of CA19-9 and clinical response in patients treated with gemcitabine and cisplatin [23]. However, they did not describe the correlation of CA19-9 response to survival and did not perform any statistical analysis. *Saad et al *demonstrated a significant correlation between the pretreatment serum CA19-9 levels, CA 19-9 response and OS in 28 patients with advanced pancreatic cancer treated with gemcitabine. In their multivariate analysis, they found that both baseline and CA19-9 response correlated to OS (p = .0005 and .0497, respectively) [24]. Similarly, in a series of 43 patients, *Halm et al *showed a significant higher OS for patients with a greater than 20% decrease in CA19-9 values from baseline after treatment with gemcitabine [25]. In this analysis, CA19-9 responses were the strongest predictor of OS. *Ueno et al *have performed a retrospective study of 103 patients with metastatic disease treated with systemic chemotherapy. In their report, serum C-reactive protein ≥ 5 mg/dL, PS of 2 or 3 and CA19-9 above 10,000 U/mL correlated significantly to shorter OS after a multivariate analysis [22]. These results suggest prognostic and predictive value of both baseline and changes in post treatment CA19-9 for OS in patients with metastatic pancreatic cancer.

Because only six patients had a surgical resection, we could not address the impact of margins and involvement of lymph nodes on length of survival. Six patients with 1 to 7 positive nodes had an OS of 13.1 to 25.0 months after recurrence. In addition, 4 patients with positive margins showed an OS of 17.7 to 25 months after recurrence. Thus, occasionally patients with positive lymph nodes or margins at the time of resection can have survival durations up to two years. Although the difference in OS between patients with well and moderately differentiated tumors compared to those with poorly differentiated tumors did not reach statistical significance, there were only 12 patients with available information on histological grade, which might limit the power to detect smaller differences. Among resected cases, only one tumor measured less than 3 cm, all patients had perineural extension, 83% had positive lymph nodes and 66% had positive surgical margins, all features of aggressive behavior and poor prognosis [26-29]. Based on this study, no histopathological characteristics correlated with OS.

Currently, there is no universally accepted standard second-line chemotherapy after gemcitabine failure for metastatic pancreatic cancer although a 5-FU (or capecitabine) based regimen would be most commonly used. We demonstrated that in a selected subgroup of patients, median TTF on gemcitabine and OS were 11.5 and 26.9 months, respectively. Therefore, median intervals greater than 1 year between gemcitabine withdrawal and death can be expected in these cases. In this study, 10 patients (50%) received at least a second-line treatment and the median number of chemotherapeutic regimens or experimental therapy in patients receiving more than one regimen was 3. These data suggest that a significant number of patients who survive more than 1 year with metastatic pancreatic cancer will still be candidates for further therapies after gemcitabine failure. Future trials of second-line therapies for this selected population seem to be warranted.

Although gemcitabine is a well-tolerated chemotherapeutic agent with a favorable toxicity profile, long-term use of gemcitabine is associated with potential development of HUS and other uncommon toxicities, as shown in this and our previous study [30]. This highlights the importance of continued monitoring for HUS and other side effects for patients undergoing prolonged treatment with gemcitabine.

## Conclusion

A subgroup of patients with metastatic pancreatic cancer treated with gemcitabine has a significantly better outcome than most patients. Besides CA19-9 and total bilirubin, no clinical or pathologic features correlated with duration of survival in these highly selected patients. Continued investigation of key molecular markers that might be capable of predicting prognosis and treatment response in pancreatic cancer patients is needed in future clinical trials. Given the relatively long median survival of this group of patients after disease progression on gemcitabine, additional systemic therapies after gemcitabine failure may improve the clinical outcomes for selected patients with metastatic pancreatic adenocarcinoma.

## Competing interests

The authors declare that they have no competing interests.

## Authors' contributions

BHLG contributed to study design, data collection, data interpretation, manuscript writing. JWC contributed to data interpretation, manuscript review. GYL contributed to description of pathology findings, manuscript review. DPR contributed to data interpretation, manuscript review. NG contributed to study case identification, manuscript review. AM contributed to statistical analysis, manuscript review. AXZ contributed to study concept, study design, data interpretation, manuscript writing and review, overall supervision.
